# Preparation and Application in Water Treatment of Magnetic Biochar

**DOI:** 10.3389/fbioe.2021.769667

**Published:** 2021-10-25

**Authors:** Qingshuang Zhao, Ting Xu, Xueping Song, Shuangxi Nie, Sun-Eun Choi, Chuanling Si

**Affiliations:** ^1^ Tianjin Key Laboratory of Pulp and Paper, Tianjin University of Science and Technology, Tianjin, China; ^2^ Guangxi Key Laboratory of Clean Pulp and Papermaking and Pollution Control, College of Light Industry and Food Engineering, Guangxi University, Nanning, China; ^3^ Department of Forest Biomaterials Engineering, College of Forest and Environmental Sciences, Kangwon National University, Chuncheon, South Korea

**Keywords:** magnetic biochar, water treatment, adsorption, magnetic response, adsorption mechanism

## Abstract

This paper reviews the preparation of magnetic biochar and its application in wastewater treatment, and briefly discusses the adsorption mechanism of biochar to remove pollutants and the modification methods of biochar. Due to the good physical and chemical properties of biochar, including its rough porous structure, it has been widely used to absorb pollutants from water. Magnetic biochar is commonly prepared by combining biochar with magnetic material. The biochar is endowed with the characteristics of the magnetic material, which could effectively solve the problems of difficult recovery and easy loss of adsorbent in water treatment. Magnetic biochar with high carbon content, large specific surface area, magnetic separation, and other excellent properties, has become a hot research topic in recent years. The preparation methods and application properties of magnetic biochar are reviewed. The future research directions of magnetic biochar are put forward to provide directions for further research and application of magnetic biochar materials.

## Introduction

In this era of rapid development of the industry, people’s needs are constantly increasing to ensure the conditions of cost control to improve the profits of the industry (Yan et al., 2021; Pei et al., 2020a; Dai et al., 2020; Li X. et al., 2019; Si et al., 2009; Yang et al., 2019; Chen et al., 2020a; Liu et al., 2020a). Agricultural waste has received extensive attention due to its wide application and availability (Si et al., 2013; Dai L. et al., 2019; Du et al., 2019; Pei et al., 2020b; Liu et al., 2021c; Liu. et al., 2021e; Ma et al., 2021; Zhang et al., 2021). In these respects, some countries, such as Malaysia, dominated by the department of agriculture, support a most economic turnaround with annual production of more than two million tons of agricultural waste (Yang et al., 2020; Liu et al., 2019; Wang et al., 2019). However, this production is commonly conducted in the open-air, leading to waste being incinerated or dumped in landfill, which can lead to serious environmental problems, such as groundwater pollution or air pollution (Si et al., 2008; Liu et al., 2020b). Materials such as empty fruit bunches, rice husks, and coconut shells were found to be some of the most abundantly available agricultural wastes which contain high amounts of minerals such as silica, magnesium, and potassium along with a high porosity (Laine and Calafat, 1989; Mubarak et al., 2014; Vadivelan et al., 2005; Mubarak et al., 2016; Chen et al., 2020b; Ma et al., 2020; Du et al., 2021).

These agricultural and forestry wastes can be formed into biochar through a common pyrolysis process (Xu, R. et al., 2020b; Cheng, et al., 2020). For example, enhancing the soil fertility for a higher crop production by increasing the fertilizer’s retention time (Liang et al., 2006; Chan et al., 2008), as a dopant to increase the capacitance value of a supercapacitor (Gupta et al., 2015), and as an adsorbent in the removal of various wastewater’s contaminants such as lead (Liu and Zhang, 2009), zinc (Chen B. et al., 2011), and natural organic matter (Uchimiya et al., 2010). If the biochar is separated in an aqueous solution, it must be centrifugated (Mukherjee et al., 2011) or activated with a strong base before the biochar can be used in a supercapacitor (Goda et al., 2014; Das et al., 2015). The development of magnetic biochar has overcome this problem, and has been applied in various fields by attaching various metal ions to the surface of biochar to make it magnetic. Magnetic biochar is prepared in the laboratory using conventional heating in an electrical furnace (Zhang et al., 2007; Theydan and Ahmed, 2012; Wang H. et al., 2020), microwave heating in a modified furnace or oven (Wang et al., 2013; Mubarak et al., 2014; Ruthiraan et al., 2015), co-precipitation (Saravanan et al., 2012; Jiang et al., 2015; Yu et al., 2013), and calcination (Gao et al., 2015; Ma et al., 2015). These magnetic biochars adsorb waste water effectively, removing pollutants, such as cadmium (Reddy and Lee, 2014; Mohan et al., 2014), arsenic (Akin et al., 2012; Gao et al., 2015), lead (Wang et al., 2015a; Wang et al., 2015b; Wang et al., 2015), methylene blue (Theydan and Ahmed, 2012; Zhang and Gao, 2013; Mubarak et al., 2014), and phosphate (Chen X. et al., 2011). Besides that, certain selected magnetic biochars showed the excellent capability to be used as electrodes for supercapacitors to increase the capacitance and electrical conductivity value as well (Li and Liu, 2014; Liu et al., 2021a; Xu T. et al., 2021; Du et al., 2022).

**FIGURE 1:**
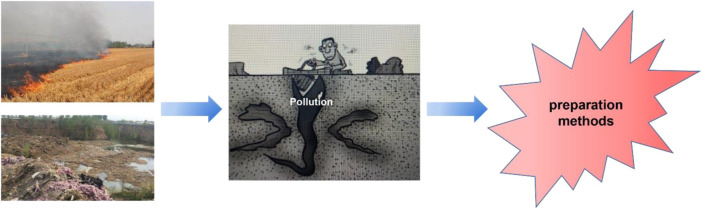
Changes in environmental patterns and solutions to problems.

This article mainly introduces the preparation method of magnetic biochar and its application in water treatment as well as its wide application in other areas. As shown in Figure 1, people throw away rubbish at will, which causes serious environmental pollution (Lin et al., 2020; Liu et al., 2021b). Water resources have also been severely damaged, but the preparation of magnetic biochar can absorb pollutants in the water. Researchers preparing magnetic biochar need to pay attention to its shortcomings, correction methods, and so on. The preparation of biochar by combining magnetic materials with biochar has become a hot topic for scientists. According to these conditions, the research targets for the future development of magnetic biochar are determined.

## Preparation Methods of Magnetic Biochar

### Pyrolysis

In general, pyrolysis refers to the thermal decomposition of organic matter in the absence of oxygen at temperatures over 400°C (Maschio et al., 1992; Thines et al., 2017). Three substances are produced, which consist of a residual solid biochar, a liquid product commonly known as bio-oil, pyrolysis oil, or biocrude, and a non-condensable gas known as syngas, which consists of carbon monoxide (CO), carbon dioxide (CO_2_), hydrogen (H_2_), and methane (CH_4_). The pyrolysis process is generally divided into two stages. In the first stage, heat is transferred to the surface of ions by radiation, from the surface to the interior. During the main pyrolysis and preheating solution, the volatiles and syngas flow through the particles in the void due to the loss of biomass water due to the increase of temperature. The process of heat transfer is a function of time. The second stage of the pyrolysis process begins with the expansion of the solid voids and the conversion of biomass into gas (Blasi, 2008). The expansion of pores provides a site for volatile gases produced during the pyrolysis process. There are three main mechanisms for heat transfer, conduction inside the biomass particle, convection inside the pores of the biomass particle, and the convection and radiation from the surface of the final product.

**FIGURE 2:**
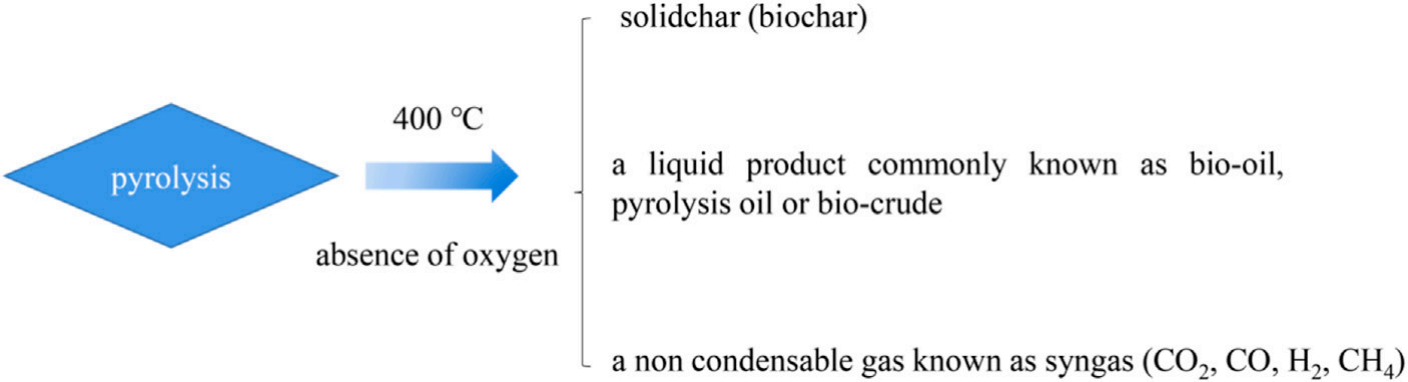
The process of thermal decomposition.

Accordingly the different operations can be divided into three types: conventional pyrolysis, fast pyrolysis, and flash pyrolysis, as shown in Table 1. The heating rate of traditional pyrolysis is relatively slow which allows for the production of solid, liquid, and gaseous products in compelling portions (Goyal et al., 2008). Traditional pyrolysis can be divided into intermittent pyrolysis and continuous pyrolysis. In the latter stage, few internal repeats occur, such as the breaking of bonds, the appearance of free radicals, the elimination of water, and the formation of hydrogen peroxide (Shafizadeh et al., 1982). The main pyrolysis mainly takes place in the second stage of the high-rate decomposition of biomass. The third stage is the slow decomposition of the carbon to form a carbon-rich residue known as biochar. The overall yield of conventional pyrolysis would be approximately 35% biochar, 30% bio-oil, and 35% syngas by mass (Laird et al., 2009). Bio-oil mainly exists in the state of steam and aerosol, and cannot be separated from syngas. If this gas is discharged into the environment, it will cause serious environmental pollution. In contrast, when production is mainly concentrated on liquid or gas products, rapid pyrolysis is chosen (Liu et al., 2021d; Liu W. et al., 2021). In the process of rapid pyrolysis, high operating temperature, short contact time, fine particles, and other operating conditions are required if rapid heating is desired (Babu, 2008). The overall yield of fast pyrolysis would be approximately 50–70% bio-oil, 10–30% biochar, and 15–20% syngas by mass (Babu, 2008). Raw materials need to be dried and ground to a size less than 2 mm before entering the main system of the pyrolysis reactor. However, the temperature of rapid pyrolysis increases rapidly and the particles are sparse. So rapid pyrolysis to produce gaseous products is a good choice (Demirbas et al., 2002). The overall yield of flash pyrolysis would be approximately 60% biochar and 40% volatiles by mass. This process consists of a gasifier which allows a small, limited amount of oxygen to enter the reaction chamber which causes partial combustion of biomass, producing 5–15% char and traces of bio-oil which are referred to as “tar” (Laird et al., 2009). As for laboratory scale, the pyrolysis process is generally done either through conventional heating or microwave heating. The product decomposition diagram is shown in Figure 2.

**TABLE 1:** Operating conditions for different types of pyrolysis processes.

Parameters	Fast pyrolysis	Flash pyrolysis	Conventional pyrolysis
Particle size (mm)	Less than 1	Less than 0.2	5–50
Residence time(s)	0.5–1.0	Less than 0.5	450–550
Heating rate (K/s)	10–200	Less than1000	0.1–1.0

### Co-Precipitation

Co-precipitation is the process by which a solute is separated from a solution by means of a carrier, by which the solute is bonded to the solute rather than dissolved in the solution. The solute is called an impurity. This kind of co-precipitation is used in purifying solution and industrial wastewater (Xu R. et al., 2021). A solute can be co-precipitated out from a solution through three common methods such as inclusion, occlusion, and surface adsorption (Kolthoff, 1932). Inclusion or also known as the formation of mixed crystals is a process which consists of the incorporation of solute in the crystal lattice of the carrier through a hole in which the regular structure of the carrier remains unchanged. This combination forms a mixed crystal in which the amount depends on the adsorption phenomena during the precipitation process.

Apart from that, during the occlusion process, the solute remains incorporated into the crystal lattice of the carrier, instead of the carrier being completely surrounded by the solute during the formation of the crystal matrix, entrapping the solute from returning to the solution. The surface adsorption process on the other describes how the solute is attached to the surface of the carrier and moves out from the solution only when the crystal matrix forms a large surface area and behaves like a flocculated colloid (Wang P. et al., 2020). In general, the precipitation process is a phenomenon that occurs when a substance reaches a certain saturation, followed by the slow growth of the nucleus as the solute diffuses to the surface of the crystal (Sugimoto, 2003). In addition, some organic ions or polymer surface complexing agents can be added to control the size of the magnetic material (Cai and Wan, 2007). Due to the simplicity of the co-precipitation process and the wide range of operating parameters, the required particle size and characteristics can be obtained for the production of magnetic biochar. Massart et al. (Massart, 1981) were first to perform a controlled preparation of superparamagnetic iron oxide particles utilizing the alkaline precipitation of FeCl_3_ and FeCl_2_ which managed to produce spherical magnetite particles with a diameter in the nanometer scale. In addition, another study by Babes et al. (Babes et al., 1999) showed that if the ratio of Fe^2+^ to Fe^3+^ increased, the particle size of the magnetic biochar increased, but the yield of magnetic biochar decreased (Wang et al., 2021). The extensive growth of this simple yet significant method which controls the particle size of magnetic particles being produced provided space for researchers to perform studies on the production of magnetic biochar based on various biomasses. In this regard, Saravanan et al. (Saravanan et al., 2012) discovered diamond glue, took out a little biological sample, and co-precipitated the ammonia solution (2:1) with Fe^2+^ and Fe^3+^ to produce magnetic iron oxide particles. This magnetic material had good thermal stability compared with the star polymer containing iron oxide particles. Similarly, Mohan et al. (Mohan et al., 2011) prepared magnetic biochar from almond shells by chemical precipitation (FeCl_3_ and FeSO_4_). The magnetic biochar had a spongy porous structure and the non-magnetic biochar had a porous shape. These remarkable characteristics provide a good source of magnetic biochar with good adsorption properties.

### Reductive Co-Deposition

The reduction co-precipitation process is similar to the co-precipitation process, but the difference is that the transition metal is reduced by a reducing agent such as sodium borohydride or potassium borohydride in the process of binding to the biochar (Wang P. et al., 2020). When the reaction is terminated, the supernatant is removed and the residue is cleaned and dried in a true empty tank to achieve the magnetic biochar. Interestingly, this material is composed of nanoparticles and most of the zero-valent metals, making the magnetic biochar produced highly reductive, and also highly effective at contaminant removal. For example, Zhu et al. (Zhu S. et al., 2018) found that the Cr adsorption capacity of reduced co-precipitated magnetic biochar was 58.82 mg/g, and that most of the Cr (VI) was reduced to Cr (III). Therefore, this method can synthesize magnetic biochar with a better effect and stronger ability to remove pollutants. However, the used reducing agent is harmful and will produce hydrogen during the reduction process, which will pose a certain risk to safety when used on a large scale.

### Hydrothermal Carbonization

Hydrothermal carbonization refers to the heterogeneous reaction of biomass and metal ions, because the reaction temperature (100–300°C) is relatively low, the reaction pressure is generated by the reaction itself (An et al., 2019). These conditions are milder than the previous conditions, there is no need to add bases or strong reductants, making the reaction easier. For example, Zhang et al. (Ngarmkam et al., 2011; Bastami and Entezari, 2012) successfully synthesized magnetic biochar by this hydrothermal method from iron-containing sludge and sludge at 473°C, and used the resulting product as a Fenton-like catalyst to completely degrade methylene blue. Similarly, Nethaji et al. (Nethaji et al., 2013) found that magnetic biochar synthesized using this method had a maximum adsorption capacity for Cr (VI) of up to 142.86 mg/g, which was greater than that of most magnetic biochar prepared by co-precipitation (Baig et al., 2014), reductive co-deposition (Devi and Saroha, 2014), or impregnation pyrolysis (Quan et al., 2014).

### Other Preparation Strategies

Recently, other methods for preparing magnetic biochar have also been developed, such as ball milling, in which biochar is mechanically mixed with iron oxides in a solvo-free manner (Shang et al., 2016), direct pyrolysis of biomass/metal salts, and cross-linking of biochar and iron oxides (Mojiri et al., 2019). For example, Shang et al. (Shang et al., 2019) found that the adsorption capacities of magnetic biochar synthesized by ball-milling of biochar and iron oxide for the pharma-ceuticals carbamazepine (CBZ) and tetracycline were 62.7 mg/g and 94.2 mg/g, respectively. In addition, Dai et al. (Dai S.-j. et al., 2019) demonstrated that the herbicides dichlorophenol and atrazine were very efficiently removed by magnetic biochar synthesized by the molten salt method.

**FIGURE 3:**
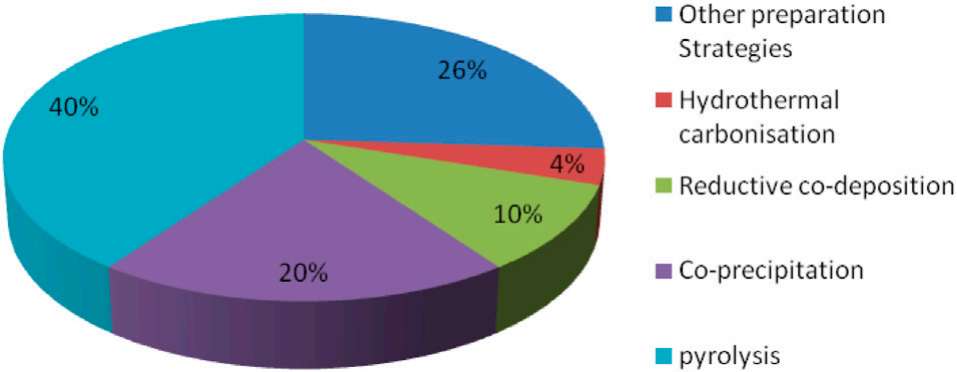
Magnetic biochar synthesis methods.

To improve magnetic biochar’s ability to heal in the environment more quickly, the researchers treated it with metal or acid or alkali solutions, and also reacted it with different chemical functional groups, in order to improve adsorption selectivity and capacity. A graphical summary of these various methods for the synthesis of magnetic biochar is shown in Figure 3.

Hydrothermal carbonization, reductive co-deposition, co-precipitation, pyrolysis, and other synthetic methods are also used to obtain magnetic biochar. Therefore, the nature of the raw material, the physicochemical properties of the pollutants, and the method operability should be carefully considered when selecting a synthetic approach for magnetic biochar.

## Application of Magnetic Biochar

Magnetic biochar has the potential to be used in purifying the environment, specifically it can be used as an adsorbent, catalyst, soil remediation agent, etc. The need for clean water is a necessity in every part of the world. The main cause of water pollution may be untreated discharge of heavy metals into nearby water sources by mining, batteries, metal plating, or hazardous wastewater such as organic matter and dyes from the textile industry (Li et al., 2020). Therefore, in order to provide clean water for users, a variety of wastewater treatment technologies are chosen such as ion exchange, membrane filtration, biological treatment, and adsorption to solve this problem.

### Adsorbent

#### Adsorption of Heavy Metals

Heavy metals in the environment also have different states. According to the characteristics of these states, heavy metals are classified as anion or cation type. The adsorption capacity of magnetic biochar for chromium is between 8.35 mg/g to 220 mg/g, which proved that the adsorption performance of magnetic biochar was greatly affected by raw materials (Ifthikar et al., 2017). The mechanism by which Cr (VI) is removed by magnetic biochar involves electrostatic adsorption, reduction, ion exchange, complexation with functional groups, and co-precipitation. The adsorption capacity of magnetic biochar is different with the difference of valence, being 1.305–45.8 mg/g and 1.630–10.07 mg/g. For the removal of As, the adsorption mechanism of magnetic biochar is mainly electrostatic adsorption and functional group complexation, among which iron oxide plays a key role (Lu et al., 2004).

The cationic heavy metal pollutants removed by magnetic biochar are mainly Cd (II), Pb (II), Cu (II), Ni (II), Sb (II), Sn (II), and Hg (II), and the efficiency of their removal is influenced by their different physical and chemical properties (Sozeri et al., 2013). The removal mechanism of metal cations by magnetic biochar is as follows: 1) electrostatic adsorption; 2) ion exchange; 3) surface complexation; 4) π-π interaction; 5) internal spherical complexation; 6) hydrogen bonding; and 7) co-deposition. Magnetic biochar is also used for the removal of multiple heavy metals. In systems polluted with multiple heavy metal species, the various heavy metals compete for adsorption sites, which affects the overall adsorption behavior of the magnetic biochar.

#### Adsorption of Nuclear Waste Pollutants

**FIGURE 4:**
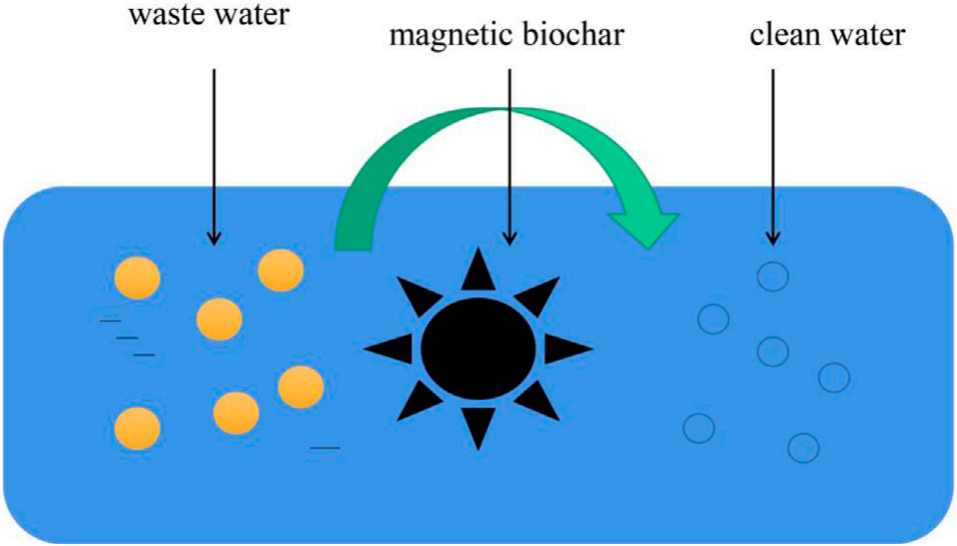
Magnetic biochar adsorbs wastewater pollutants.

In recent years, magnetic biochar has become more widely used to remove pollutants from nuclear waste U (VI) and Eu (III) (Hu et al., 2018). For example, Zhu et al. (Zhu Y. et al., 2018) found that the maximum adsorption capacity of magnetic biochar to Eu was 105.53 mg/g, and concluded that the mechanism of magnetic biochar’s removal of pollutants was surface and inner sphere complexation. At the same time, Li M. et al.‘s (Li M. et al., 2019) research results showed that the maximum adsorption capacity of magnetic biochar for U is 52.63 mg/g, and its pollutant removal mechanism is inner sphere complexation. It can be seen from these that the pollutants from nuclear waste can be effectively removed by magnetic biochar, which is a process of enrichment, when the complex charge of these pollutants in magnetic biochar is relatively high. Therefore, what to do with magnetic biochar, which contains pollutants that absorb nuclear waste, also remains a challenge. The schematic diagram of purifying wastewater with magnetic biochar is shown in Figure 4.

#### Adsorption of Organic Pollutants

Magnetic biochar showed a good adsorption performance for organic pollutants, and the removal of organic pollutants by magnetic biochar was mainly antibiotics, organic dyes, pesticides, and organochlorine compounds (Kumar et al., 2020). Meng et al. (Meng et al., 2015) summarized the adsorption effect of magnetic biochar on antibiotics. According to these data, tetracycline is the most common removal, with adsorption loads ranging from 33.1 mg/g to 297.61 mg/g, followed by methanoxazole, with adsorption loads ranging from 5.19 to 212.8 mg/g. It mainly summarized the mechanism of antibiotic removal by magnetic biochar and these involve hydrogen bonding, π-π interactions, pore-filling effects, electrostatic adsorption, and hydrophobic interactions.

The organic dyes that are adsorbed by magnetic biochar are rhodamine B, methylene blue, malachite green, acid orange 7 (AO-7), and orange-G. The saturated adsorption capacity of magnetic biochar for these dyes ranged from 31.25 to 388.65 mg/g, while the mechanism of adsorption of dyes by magnetic biochar was less discussed.

At the same time, magnetic biochar can also remove other organic pollutants, such as pesticides, phenol, organochlorine, and hormones. The adsorption capacity varies from 3.46 to 169.7 mg/g. The removal mechanisms of these substances by magnetic biochar involve hydrogen bonding, π-π interactions, pore-filling effects, electrostatic adsorption, hydrophobic interactions, and reductive dehalogenation (Mun et al., 2013).

#### Adsorption of Inorganic Anion Pollutants

**FIGURE 5:**
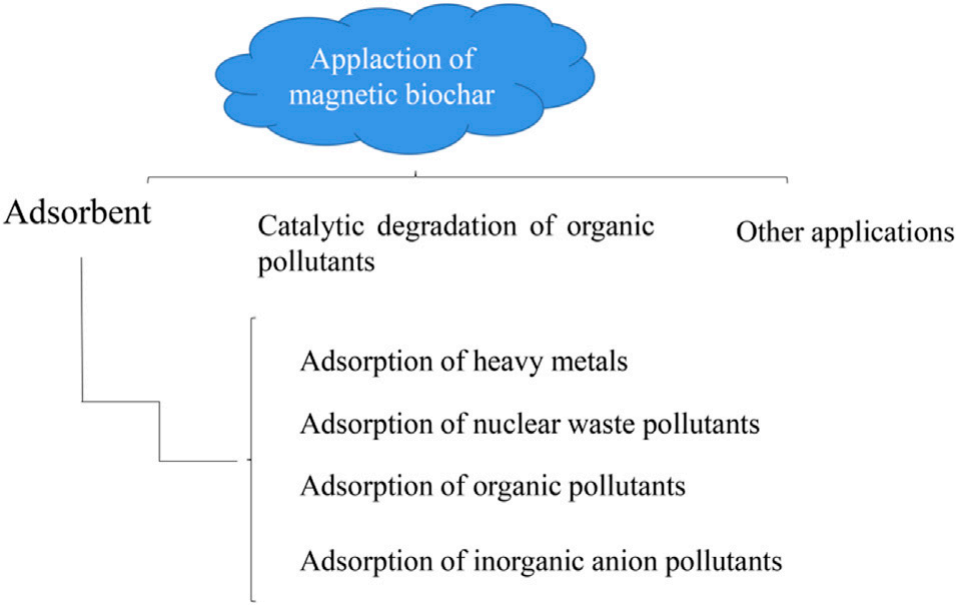
The removal of antibiotics by magnetic biochar.

Magnetic biochar can also be used to remove inorganic pollutants such as phosphate, nitrate, fluoride, etc. (Cai et al., 2017; Xu et al., 2020a). Compared with other pollutants, nitrate has been studied the most, and the adsorption capacity of magnetic biochar is between 1.26 and 474.26 mg/g (Li et al., 2017). The modified magnetic biochar has a good adsorption capacity of phosphate. In addition, the magnetic biochar has a good adsorption capacity of nitrate and fluoride, and the adsorption capacity is about 9 to 15 mg/g. The results show that co-precipitation, electrostatic adsorption, surface complexation, inner sphere complexation, and ligand exchange are present. Finally, due to the ability of magnetic biochar to adsorb a large number of nutrients, it could be used as a slow-release fertilizer to improve the composition of soil elements and soil fertility (Figure 5).

### Catalytic Degradation of Organic Pollutants

Magnetic biochar has also been used as a catalyst for hydrogen peroxide, producing a highly reactive oxidizing substance that is used to degrade organic matter. For example, Chen et al. (Chen et al., 2018) found that the antibiotic ofloxacin could be efficiently degraded by magnetic biochar and persulfate. Meanwhile, Zhang et al. (Zhang et al., 2018) demonstrated that methylene blue can be completely removed by magnetic biochar coupled with hydrogen peroxide. Because different organisms have different physical and chemical properties, some organisms cannot remove organic pollutants at all by using female organisms. So, we can take advantage of the function of the female organism, the catalytic property, which is to produce hydroxyl radicals inside of the organism in time, to produce strong oxidizing radicals that attack organic pollutants freely, in a way that maximizes the function of the magnetic biochar. If it is difficult to remove organic pollutants by using magnetic biological carbon, its catalytic properties can be considered to achieve efficient degradation of organic pollutants.

### Other Applications

In addition to the above functions, magnetic biochar can also be used as a photocatalytic carrier, for example, Li M. et al. (Li M. et al., 2019) prepared the Fe_3_O_4_/BiOBr/BC photocatalyst using magnetic biochar as a carrier and found that its removal efficiency of propranolol was nearly 95%. Similarly, magnetic biochar can effectively recover precious metals by adsorption and enrichment. For example, Zhang et al. (Zhang et al., 2019) found that the adsorption capacity of magnetic biochar for silver ions was 818.4 mg/g.

It is very interesting that Qin et al. (Qin et al., 2017) used magnetic organisms in the process of anaerobic digestion, but as an additive, they found that a small amount of magnetic biochar promoted the production of methane on anaerobic digestion, and finally found methanogenic bacteria that produced methane, which facilitated recycling. And finally, female organisms could not just be used for environmental remediation, but also for energy storage (Hu et al., 2019; Lu et al., 2019). For example, Thines et al. (Thines et al., 2017) found that the density capacitance and energy could be increased when magnetic biological carbon was used as an electric conductor. Meanwhile, Jiang et al.‘s (Jiang et al., 2019) research results showed that hydrogen could be effectively recovered in the process of removing nitrophenol. Although magnetic biochar has a good adsorption effect, due to the release of a lot of heat in the preparation process, the yield is low, and the adsorption may not be complete, difficult to recover, and may cause secondary pollution to the environment. The large-scale application of magnetic biochar is limited.

## Conclusion and Prospects

The remarkable properties of magnetic biochar and its special role in related industrial production have attracted great attention. In fact, demand for magnetic biochar has been growing over the past few years. However, this extensive demand is faced with the innovation and breakthrough of the preparation technology, and the solution of the current key technology and restriction links is conducive to the further large-scale production of magnetic biological carbon. In fact, the successful experience gives people a profound enlightenment, one to optimize the choice of production materials. As mentioned earlier, a large amount of agricultural waste can be used to produce magnetic biological carbon, and the expansion potential is huge. Second, the production process should be selected reasonably. For the production of magnetic biochar from agricultural wastes, new processes should be established based on actual conditions. In terms of selection criteria, not only the characterization elements of raw materials should be considered, but also important factors such as moisture content, ash content, carbon content, and surface area should be fully considered. These elements have a direct effect on the morphology and properties of the produced magnetic biochar, and will play a crucial role.

Therefore, the production of magnetic biochar from agricultural waste must be thoroughly understood and analyzed before it is developed into magnetic biochar. In addition, agricultural wastes must be pre-treated with particular care to avoid the spread of harmful pollutants from these wastes and their spread into the surrounding environment through water or air sources. At the same time, it is necessary to fully consider the transportation cost of batch scale and the cost of pretreatment when producing magnetic biochar from various agricultural wastes. It is very important to choose an open site for the treatment of agricultural waste. Through the combustion treatment, the prevention and control of gas pollution should be fully considered. The negative impact on the surrounding environment can be effectively reduced during the production of magnetic biochar through the application of new processes. Magnetic biochar can be produced by new environmental protection technology and strive to become an environmentally friendly product. On the other hand, after the successful development of magnetic biochar, the problems of marketing must be fully considered. The production of magnetic biochar from agricultural waste and its application in wastewater treatment or the electrolysis industry need a process of application and effect approval. It is necessary to organize effective verification tests, especially to carry out cooperation between science and enterprise, and to carry out series promotion and application in combination with production practice. If magnetic biochar products are successful in specific applications, more enterprises will invest in this environmental protection industry, which will further promote the development of the magnetic biochar industry for agricultural waste development. At the same time, the application of magnetic biochar can be expanded by coating other carbon materials such as carbon nanotubes or graphene or polymers to improve its performance and expand its application in other industries.

In fact, the wide application of biochar in different industries requires continuous improvement, and research and development of new magnetic biochar production is very necessary. It is undoubtedly a new attempt to produce magnetic biochar with specific properties by improving the production method and improving the adsorption characteristics of biological carbon. Three main production methods have been identified as successful in producing effective forms of magnetic biochar with high porosity, magnetic strength, and significant morphology, which can perform well in related applications. It has been proved that magnetic biochar produced from various types of agricultural wastes has a high adsorption capacity to lead, chromium, copper, tetracycline, methylene blue, and crystal violet. In addition, magnetic biochar loaded with different metal salt ions also has high capacity, high density, and high efficiency. Therefore, the production of magnetic biochar by different production methods can open up a new recycling method for the full utilization of abundant agricultural wastes, and its application prospect is very broad.
